# Bosworth fracture-dislocation of the ankle: a case report and literature review

**DOI:** 10.3389/fsurg.2026.1783662

**Published:** 2026-04-14

**Authors:** Shun Lin, Hang Zhuo, Yan Gong, Xueqiang Shen, Xiaochun Li, Zhiqiang Wang

**Affiliations:** 1 Department of Orthopaedics, Suzhou TCM Hospital Affiliated with Nanjing University of Chinese Medicine, Suzhou, China; 2 Department of Orthopaedics, Nanjing University of Chinese Medicine, Nanjing, China

**Keywords:** bosworth fracture-dislocation, case report, floating position, fracture-dislocation diagnosis and treatment, imaging features, irreducible ankleinjury, open reduction and internal fixation

## Abstract

**Introduction:**

Bosworth fracture-dislocation is a rare and specific variant of ankle injury. Its insidious radiographic features frequently lead to a high clinical misdiagnosis rate. Furthermore, the entrapment of the proximal fibular fracture fragment behind the posterolateral tibial ridge or the posterior malleolar fragment makes closed reduction exceptionally difficult.

**Case presentation:**

This report presents the case of a 56-year-old male patient admitted with swelling and deformity of the right ankle following a fall. The diagnosis of a Bosworth fracture-dislocation was confirmed via clinical history, standard radiographs, and computed tomography imaging. The patient required surgical intervention after two failed preoperative manual reduction attempts. Initially, open reduction and internal fixation were performed via a posterolateral approach with the patient in a prone position. However, postoperative imaging revealed a failed reduction characterized by persistent proximal fibular entrapment within the posterior malleolus. Following thorough communication, a revision surgery was performed through the original incision with the patient in a floating position. This approach successfully released the entrapped fibula and reduced the distal tibiofibular syndesmosis, achieving stable fixation and ultimately leading to satisfactory functional recovery.

**Discussion:**

The failure of the initial surgery highlights the hidden entrapment pitfalls associated with this specific injury. The prone position restricts the ability to obtain true intraoperative standard lateral radiographs, increasing the risk of unrecognized persistent subluxation. Adopting a floating position effectively overcomes these limitations by providing ample spatial clearance for real-time fluoroscopic monitoring and utilizing gravity for gentle axial traction, thereby ensuring the definitive visual release of the entrapped fibula.

**Conclusion:**

Bosworth fracture-dislocation is a rare injury that is easily misdiagnosed, making early diagnosis crucial. When closed reduction proves difficult, early open reduction should be performed. Utilizing a floating position intraoperatively helps ensure adequate exposure of the fracture site and facilitates standard lateral x-rays, which is an important strategy to avoid reduction failure.

## Introduction

Ankle fracture is one of the most common fractures of the lower limbs. which is generally caused by indirect violence, and it leads to fractures and dislocations of the ankle joint. Depending on the size, direction and location of the violence suffered during an ankle injury, ankle fractures can be divided into several types. Bosworth fracture-dislocation, first proposed by David Bosworth in 1947, is a rare fracture-dislocation of the ankle where the proximal fibular fragment lodges behind the tibia with a dislocation at the ankle joint (1).

Clinically, Bosworth fracture-dislocation is concealed on an ordinary lateral x-ray, caused by misapprehension of ecumenic fracture and ankle dislocation, and senior Orthopedists sometimes have challenges identifying them. Early detection of the emptiness of tibial and fibular incisions and retropositioning of the fibula can ensure a definite diagnosis by computed tomography (CT) combined with iterative reconstruction. However, CT is not a routine examination procedure and often cannot be performed in the early stages. Consequently, early diagnosis and treatment of Bosworth fracture-dislocation is significant to carry out the normal and lateral position, oblique position and tenon position of the ankle joint and to master the specific imaging characteristics of Bosworth fracture-dislocation.

Yougun et al. (2) retrospectively analyzed 3, 140 cases of ankle fractures, including 1,589 cases of supination and external rotation fracture and 51 cases of Bosworth fracture-dislocation. The incidence of Bosworth fracture-dislocation in ankle fractures is about 1.62%, and that of posterior external rotation injury in ankle fractures is 3.21%. Karel et al. found 47 articles on 97 cases through a literature review on Bosworth fracture-dislocation from 1947 to 2018 (3). This indicates that Bosworth fracture-dislocation is clinically rare and its incidence is extremely low.

In addition, another characteristic of Bosworth fracture-dislocation is the high rate of manual reduction failure. Since the proximal fibula fracture is locked in the posterolateral ridge of the tibia or the catagmatic end of the posterior malleolus (4), conventional reduction techniques are too difficult to succeed in.

A patient with Bosworth fracture-dislocation was admitted in 2019. The patient had undergone multiple unsuccessful preoperative restorations before the surgery. The prone position was adopted during the surgery, and the posterolateral approach to the ankle joint was used. Postoperative review of CT films showed that the fibula was still inserted into the fractured end of the posterior ankle, which requires a timely secondary revision surgery. The patient's function was restored with no occurrence of complications such as parenchymal infection and fascial compartment syndrome, even though the patient had several rehabilitation and surgical trauma resulting in a poor condition of the soft tissue. In order to ensure early diagnosis, reduce the missed diagnosis rate, and improve the efficacy of clinical treatment, a summary of the experiences and lessons from the diagnosis and treatment of this patient are reported as follows.

## Case presentation

**Figure 1:**
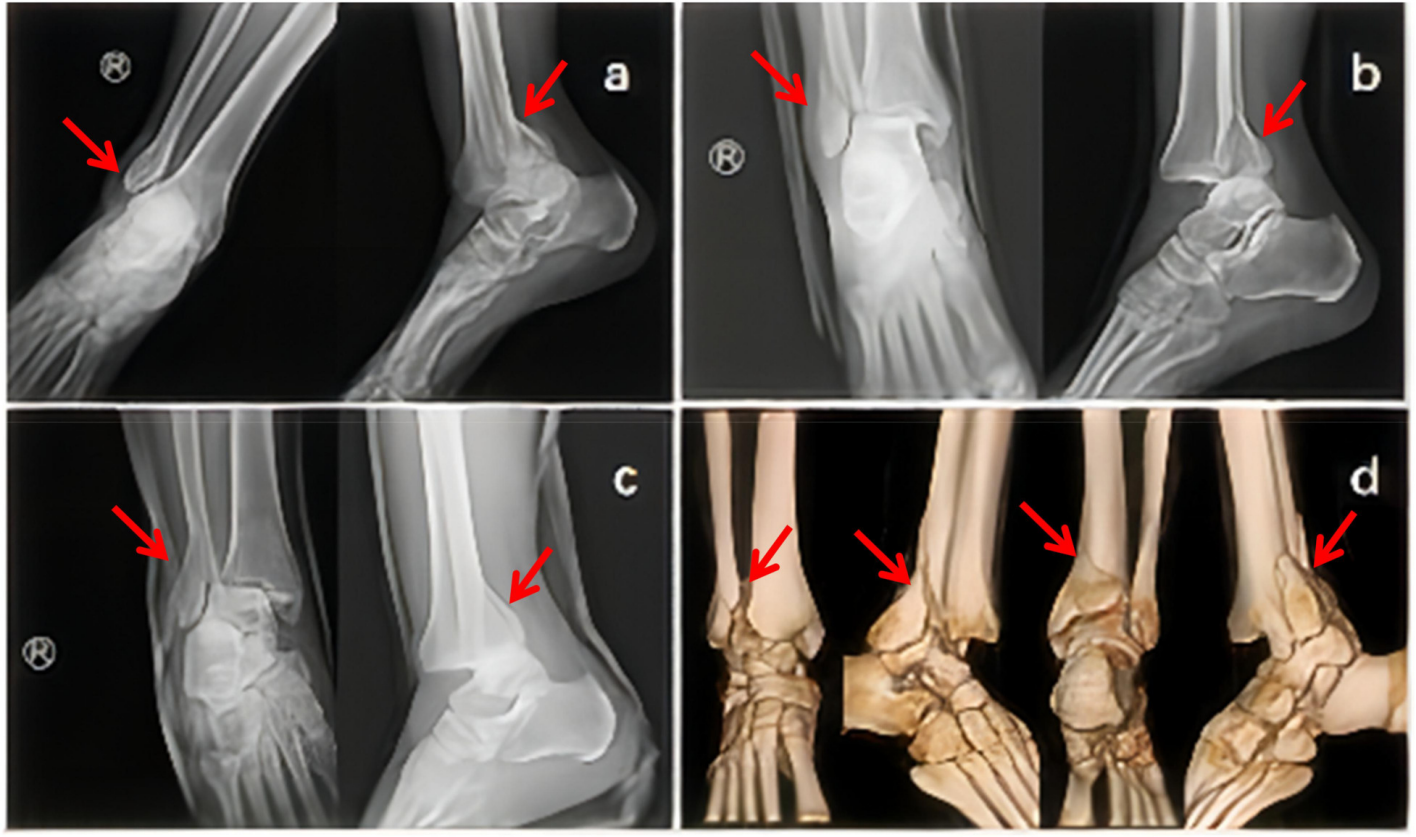
**(a)** X-ray showed fracture and dislocation of ankle joint at the time of treatment; **(b)** x-ray after the first reduction, fracture-dislocation was not corrected; **(c)** x-ray after the second reduction, fracture-dislocation was still not remedied; **(d)** the second review of CT with three-dimensional reconstruction was diagnosed as Bosworth fracture-dislocation.

A 56-year-old male patient with difficulty in walking reported to the emergency department for further treatment and presented with pain and a right swollen ankle joint due to a fall. At the time of treatment, the patient's right ankle was significantly swollen and deformed, accompanied with tenderness of the internal and external malleolus, a palpable sense of bone rubbing and normal pulsation of the dorsalis pedis artery. Routine anterior lateral x-ray examination of the ankle was performed immediately, and it indicated subluxation of the right ankle fracture (Figure 1a). After manual reduction and splint fixation, the emergency physician rechecked the x-ray, which showed that the dislocation had not been corrected (Figure 1b). One more manual reduction was attempted with a replaced polymer plaster fixation since the patient had been admitted to the hospital. However, the roentgenogram still showed dislocation at the joint (Figure 1c). CT and three-dimensional reconstruction demonstrated the images of distal fibula fracture, posterior malleolus fracture, proximal fibula fracture inserted in the fractured end of the posterior malleolus fracture, and posterior lateral dislocation of the talus, which could definitely be diagnosed as Bosworth fracture dislocation (Figure 1d). Although manipulative reduction can force the tibia into the ankle joint, it will move out of place promptly after the grip is taken off, just like the key sign of acromioclavicular joint dislocation. Immediate treatment such as calcaneal traction and mannitol dehydration should be given, considering the repeated unsuccessful reduction and the pronounced swollen limb.

**Figure 2:**
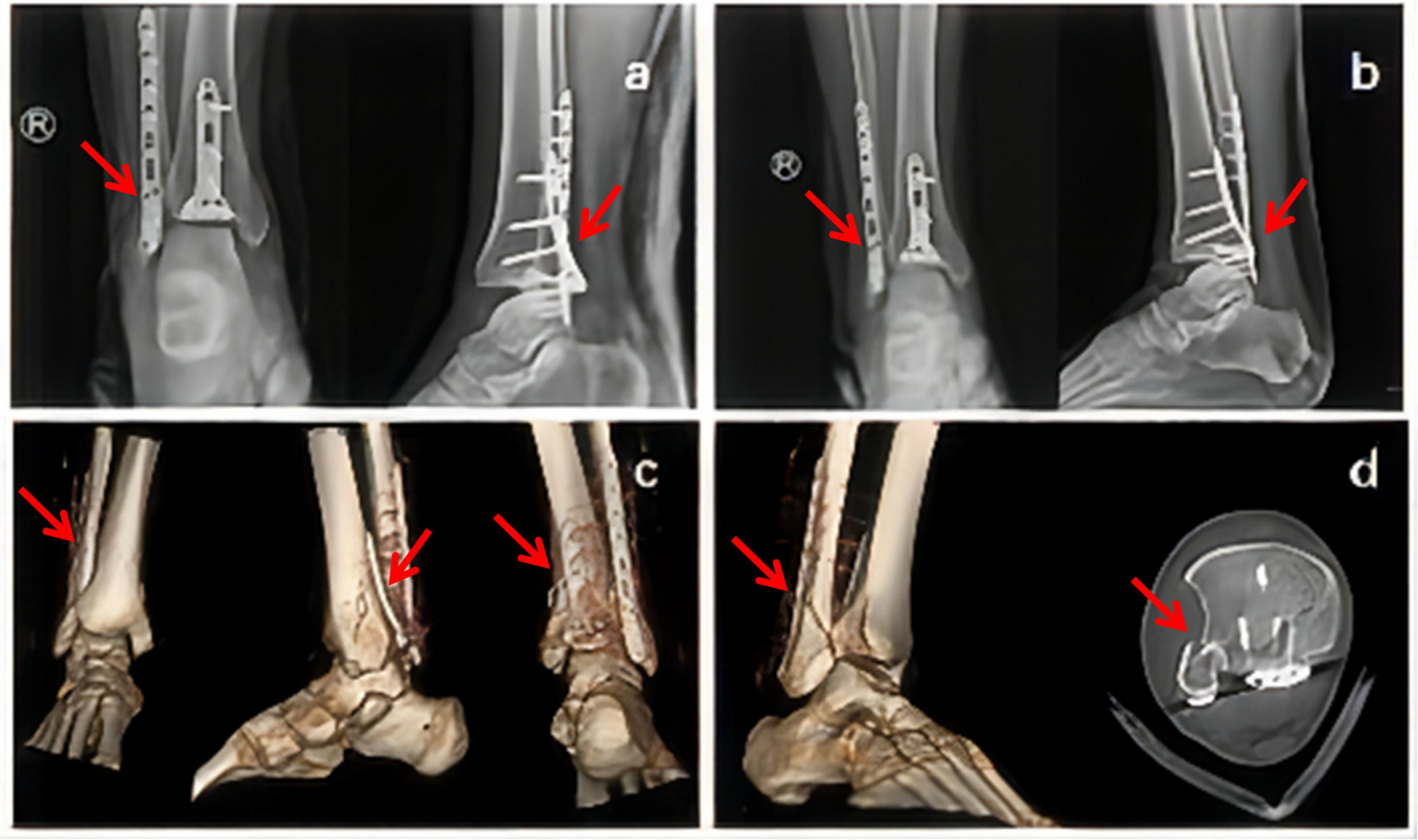
**(a)** The first review of x-ray after operation showed that the anterior space of the ankle joint is too large; **(b)** the x-ray of the post-operative dorsal flexion cast fixation showed that the anterior gap of the ankle joint decreases, but the ankle point still showed poor result; **(c)** postoperative review of CT plus three-dimensional reconstruction, Bosworth fracture-dislocation was not corrected.

One week after the patient was hospitalized, the swelling was significantly alleviated. An open reduction and internal fixation of ankle fracture and dislocation were performed, ruling out all surgical contraindications. During surgery, the prone position was adopted, and the posterolateral incision of the ankle joint was approached behind the fibular longus and brevis muscle to expose the broken end of fracture, in order to clean the intravascular bleeding and fix the broken end with the locking plate at the posterior fibula. By approaching the interstice between the fibular longus and brevis and the flexor hallux longus muscles, the posterior fracture fragment of the tibia was exposed and fixed with a T-lock plate. The reduction was achieved under direct vision, and no obvious abnormality was found in the movable ankle joint. Postoperative review of the x-ray indicated that the fractured end was well aligned, and the positive radiograph showed that the ankle point was normal, but the space between the anterior tibia and talus on the lateral radiograph was larger (Figure 2a). The mechanism is taken into consideration where the anterior joint capsule, ligament and other soft tissues are to be torn and loose. The limb was fixed at 90 degrees dorsiflexion while the plaster cast was replaced. The x-ray examination showed that the interstice was still too large (Figure 2b). Immediate CT and three-dimensional reconstruction were performed, and the fibula was not observed in the tibial and fibular notch in the transverse view. The proximal end of the fibula was still inserted in the fractured end of the posterior malleolus, and the dislocation of the tibiotalar joint remained (Figure 2c). Thus, the first surgery failed.

**Figure 3:**
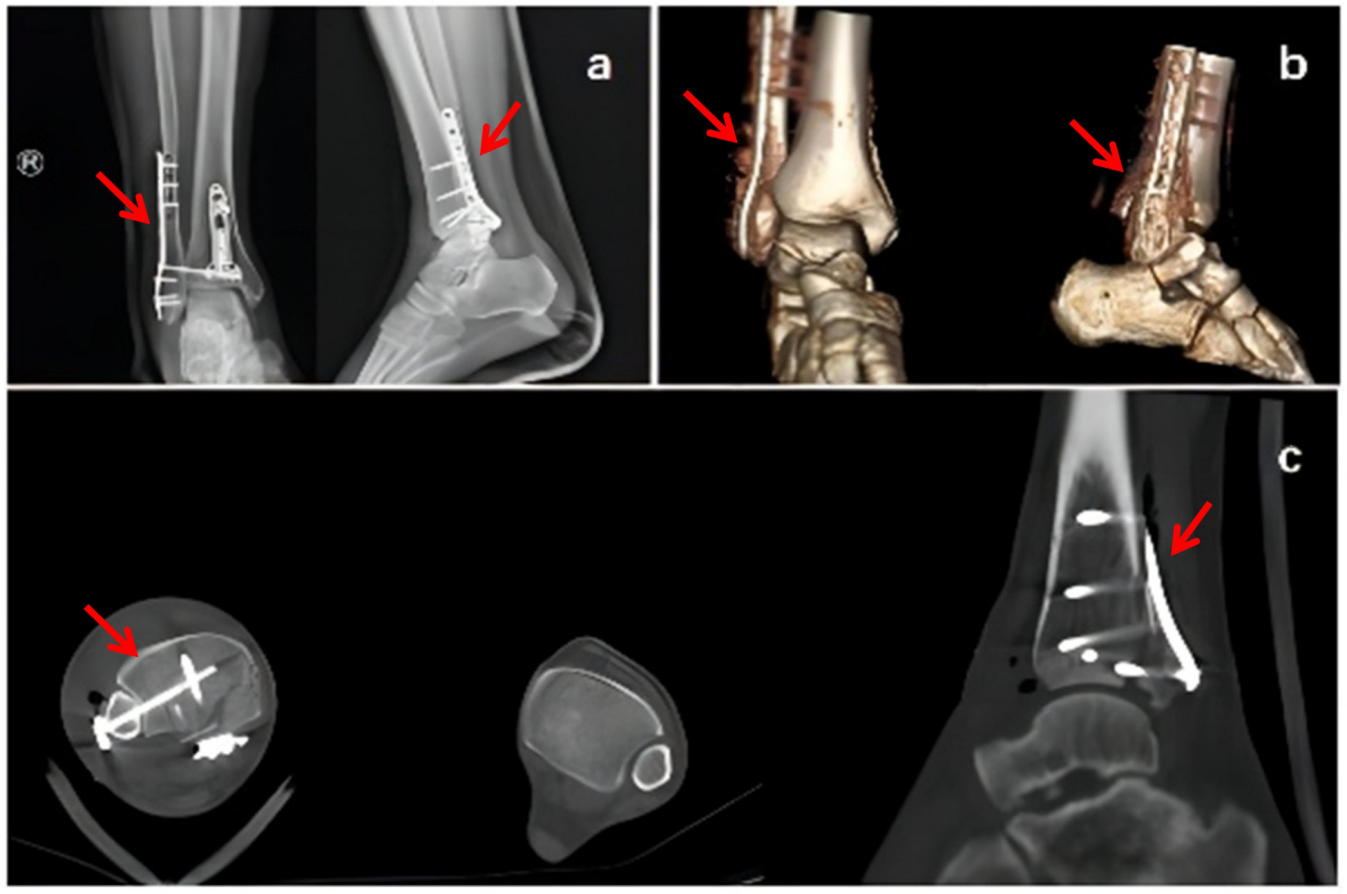
**(a)** The first review of the x-ray after operation indicates that the ankle is in place, and the alignment of the fracture can be aligned; **(b, c)** postoperative review of CT with three-dimensional reconstruction shows the Bosworth fracture-dislocation had been reset.

Full communication with the patient and his relatives, who gave the permission to carry out revision surgery. We adopted the floating position and the primary incision to expose the inferior tibiofibular syndesmosis indicating the lacerated anterior tibiofibular ligament, the dislocated inferior tibiofibular joint and the impaction. With the fibula in position, the pry was inserted in front of the proximal end of the fibula fracture. Reduction by leverage was successful after releasing the posterior tibiofibular ligament. Nickel-clad steel plate was fixed at the distal fibula to reduce the fibular fracture which resulted in a connection of the fibular notch of the tibia. Thus, the second surgery was successful. Postoperative review of the x-ray and CT with three-dimensional reconstruction showed that the fracture dislocation was corrected (Figure 3). Three months after the operation, the function of the ankle joint recovered with satisfactory effect.

## Results

A 56-year-old male presented with right ankle swelling, deformity, and limited mobility following a fall. Initial radiographs indicated an ankle fracture with subluxation, and two subsequent manual reduction attempts using splint or cast immobilization failed. Computed tomography with three-dimensional reconstruction confirmed a Bosworth fracture-dislocation. This complex injury included a distal fibular fracture, a posterior malleolar fracture, proximal fibular entrapment at the posterior malleolus, and posterolateral talar dislocation.

One week later, once the swelling had subsided and surgical contraindications were cleared, the patient underwent open reduction and internal fixation via a posterolateral approach in the prone position. Although intraoperative visualization suggested a successful reduction, postoperative imaging revealed persistent anterior tibiotalar joint widening, unresolved fibular entrapment, and ongoing tibiotalar dislocation. These findings marked the failure of the initial surgery.

Following patient consent, revision surgery was performed through the original incision with the patient placed in a floating position. Intraoperative exploration visually confirmed a lacerated anterior inferior tibiofibular ligament, dislocation of the distal tibiofibular joint, and severe bony impaction. The entrapped fibula was released via leverage reduction after dissection of the posterior inferior tibiofibular ligament. Subsequent fibular fracture fixation with a nickel-clad plate successfully restored anatomical alignment within the tibiofibular notch. Postoperative imaging confirmed the complete correction of the fracture-dislocation. At the 3-month follow-up, the patient exhibited satisfactory ankle joint function without any complications.

### Rare and high misdiagnosis ratio

Bosworth fracture-dislocation is clinically rare and presents with subtle, insidious imaging features, posing significant challenges even for senior orthopedic surgeons during immediate diagnosis. Cho et al. (5) reviewed 15 cases and found that only 5 patients were accurately diagnosed on initial imaging, reflecting a primary diagnosis rate of just 33.3%. Similarly, Yougun et al. (2) retrospectively analyzed 51 patients across four university hospitals. Their statistical analysis revealed that 22 patients were missed preoperatively and 10 were only diagnosed during surgery, yielding an initial diagnostic rate of 43. 1%. Consequently, the primary diagnosis rate of Bosworth fracture-dislocation is notably low, accompanied by a high clinical misdiagnosis rate.

To improve clinical diagnostic efficiency, a thorough understanding of the injury mechanism is essential. Schatzker et al. (6) proposed that posterolateral talar dislocation initially injures the inferior tibiofibular ligaments, leading to a fibula fracture upon further talar external rotation. Currently, most scholars agree that the injury mechanism predominantly involves severe external rotation, analogous to Maisonneuve injuries. A Bosworth lesion specifically occurs when this strong external rotational force continues relentlessly (7). Characteristic clinical signs include an ankle joint locked in extreme external rotation and profound difficulty with manipulative reduction.

Radiographically, typical diagnostic features include the “axilla sign” on the mortise view, posterior talar dislocation on the lateral view, and a specific spatial relationship between the fibula and the talus on the external oblique view (8–10). The axilla sign results from continuous internal tibial rotation, rendering the “axilla” of the medial tibial plafond visible on x-rays. Obtaining a true mortise view requires a 15-to-25-degree internal rotation projection, which is frequently unachievable clinically due to severe patient pain. It should be noted that the incarceration of tibial tendons or fracture fragments can also produce an axilla sign. Khan et al. (10) observed the axilla sign in all 10 of their retrospectively analyzed patients. Therefore, while the axilla sign alone is not an absolute diagnostic criterion, it possesses high sensitivity and specificity for predicting a Bosworth injury.

Standard lateral radiographs clearly demonstrate the posterior dislocation of the talus. Unlike common ankle fracture-dislocations where manual reduction is generally effective, Bosworth injuries resist reduction due to bony entrapment. The joint tends to re-dislocate immediately upon the release of manual pressure, a phenomenon biomechanically similar to the “piano key” sign observed in acromioclavicular joint dislocations. On external oblique x-rays, the fibula typically appears superimposed centrally on the talus. Kyu et al. (8) divided the talus into two distinct portions on this view: an anterior part (*α*) and a posterior part (*β*), separated by the fibula. Because the fibula is centrally located in a Bosworth injury, the ratio of *α*/(α+*β*) approaches 0.5. Thus, assessing the position of the fibula relative to the talus on an oblique film provides strong diagnostic evidence.

Because severe pain often precludes standard positioning and obscures these classical radiographic signs, CT with three-dimensional reconstruction is highly recommended for any suspected case. This advanced imaging allows for a prompt and clear diagnosis, minimizes the risk of further iatrogenic injury, and guides effective early surgical planning.

### Difficulty in manipulative reduction

The profound difficulty associated with reducing a Bosworth fracture-dislocation has earned it the classification of an “irreducible” ankle injury (11). The core challenge of manual reduction lies in unlocking the entrapment of the proximal fibular fragment from the posterolateral tibial ridge. Overcoming this condition requires adherence to a specific biomechanical sequence to release the locked state. The key to successful reduction hinges on restoring the anatomical alignment of the distal tibiofibular syndesmosis alongside the stability of the ankle mortise. If stress testing reveals no widening of the medial clear space and no syndesmotic separation, the joint possesses inherent post-reduction stability, making conservative treatment viable. Conversely, if instability is observed under stress, even if radiographic alignment appears adequate, conservative treatment will likely fail and mandate surgical internal fixation.

Cho et al. (5) reported an average of 2.24 manual reduction attempts across 15 cases with only 1 patient achieving successful closed reduction. Jan et al. (12) reviewed 88 cases in the literature and found only 5 successful manual reductions. Since 2011, our hospital has managed 6 such cases with only 1 successfully treated via cast immobilization. Due to the low clinical incidence of this injury, a universally accepted optimal surgical protocol is currently lacking.

In the current case, an audible clunk was heard during the initial surgery when manipulating the tibiotalar joint, giving the false impression of a successful reduction. However, the proximal fibular fragment remained firmly impacted within the posterior malleolar fracture site, which was the primary cause of the postoperative re-dislocation. This behaves much like the acromioclavicular piano key sign. The crux of intraoperative reduction is the definitive release of this fibular entrapment. Inadequate exposure during our initial surgery prevented the direct visualization required to unlock the fragment, leading to fixation failure. Therefore, surgically exposing the distal tibiofibular window for direct visual reduction is imperative to accurately restore syndesmotic anatomy and mortise stability. This direct approach sequentially ensures the proper healing of the posterior malleolus, medial malleolus, syndesmosis, and medial collateral ligament (13).

### Intraoperative position

For Bosworth fracture-dislocation, a posterolateral ankle approach is routinely recommended, as it provides optimal exposure for lateral malleolar fractures and allows for the management of posterior malleolar fractures. If only the posterolateral approach is selected, the patient is conventionally placed in a prone position. However, if there is a concurrent medial malleolar fracture, a floating position is typically adopted. For patients positioned laterally, they should lie on their uninjured side with a posterior pelvic baffle for support. When transitioning from the posterolateral approach to address the medial side, this posterior baffle can be removed to allow the patient to roll into a supine position.

In our initial surgery, the prone position was chosen because no medial malleolar fracture was identified. Following intraoperative reduction and fixation, routine C-arm fluoroscopy was performed. The intraoperative radiographs indicated a seemingly correct ankle joint position, accurate alignment of the distal tibia and fibula, and good fracture alignment. Because a true standard lateral radiograph could not be obtained in the prone position, the available lateral views only revealed an excessively widened anterior ankle space. Compounded by the severe tearing of the joint capsule and ligaments, this lack of true lateral visualization contributed to the failure of the first surgery.

In the revision surgery, utilizing a floating position combined with a posterolateral approach allowed us to reduce the fibular fracture anterior to the peroneus longus and brevis tendons. This technique yielded excellent exposure of the fracture ends and the distal tibiofibular joint. Furthermore, the floating position permits routine and unimpeded lateral ankle radiographs, which are vital for confirming reduction decisions. Another significant advantage is that the floating position allows simultaneous access to the medial malleolus without the need for re-draping, thereby saving valuable surgical time. Therefore, we strongly recommend combining the floating position with a posterolateral approach to prevent reduction failures and to safely expose posterior ankle fractures through the interval between the peroneus longus and flexor hallucis longus tendons.

Compared to the prone position, the floating posture offers substantial mechanical and operational advantages. Mechanically, it utilizes gravity to create continuous and gentle axial traction. Suspending the affected limb relaxes the musculature around the knee and ankle, effectively counteracting the shortening forces caused by post-fracture muscle contracture. This relaxation allows the surgeon to apply precise rotational or angular stress. Operationally, the floating posture provides ample spatial clearance for both surgical manipulation and C-arm fluoroscopy, ensuring real-time monitoring of reduction accuracy without obstructing the surgical field.

### Emergency surgery

Compared to conventional ankle fracture-dislocations, Bosworth injuries carry a significantly higher complication rate, including skin necrosis, wound infection, compartment syndrome, post-traumatic arthritis, avascular necrosis of the talus, and joint stiffness (7, 12, 14–18). Furthermore, these injuries are frequently accompanied by severe soft tissue damage, which can be easily exacerbated by forceful or improper manual reduction attempts. Won et al. (2) retrospectively analyzed 51 patients, comparing 36 who underwent emergency surgery with 15 who had delayed surgery. At the 1-year follow-up, the American Orthopaedic Foot & Ankle Society (AOFAS) scores for the emergency surgery group were significantly higher. Additionally, patients in the delayed surgery group exhibited persistent swelling and suffered from significantly higher rates of nonunion and osteoarthritis.

Cho et al. (5) followed 16 patients and found that those who underwent only one to two closed reduction attempts had significantly better prognoses than those who underwent three or more attempts. Furthermore, patients operated on within 24 h of injury demonstrated markedly superior outcomes. In summary, prompt diagnosis and early open reduction dramatically reduce the complication rate of Bosworth fracture-dislocation (2, 19, 20). Because of the extreme difficulty of manual reduction, the majority of scholars consider early open reduction and internal fixation to be the primary treatment of choice (2, 12, 20, 21).

The critical lesson learned from our revision surgery is the necessity of recognizing intraoperative warning signs of persistent fibular entrapment. These signs can be identified across three dimensions: biomechanics, imaging, and soft tissue tension: (1) Biomechanical deedback: if the fibular fracture fragment demonstrates elastic recoil, springing back to its displaced position when traction or temporary fixation is released, or if the ankle mortise remains widened despite compression with a reduction forceps, it strongly suggests that the proximal fibula remains locked behind the posterolateral tibial ridge. (2) Fluoroscopic features: if lateral radiographs or C-arm imaging show the distal fibula resting persistently posterior to the tibial midline, accompanied by posterior talar subluxation and the loss of the normal concentric circles of the ankle mortise, the posterior entrapment has not been completely resolved. (3) Abnormal soft tissue tension: difficulty approximating the skin edges at the posterolateral incision due to excessive tension, or significantly elevated tension upon palpation of the anterolateral fascial compartments of the leg, points to progressively worsening deep hematoma or tissue edema.

Given the high-energy nature of Bosworth fracture-dislocation, meticulous intraoperative protection of the skin and soft tissues is paramount. Compared to traditional plate fixation alone, modified techniques combining a lateral fibular plate with posterior malleolar lag screws prove highly beneficial for unlocking the fibula and securely reducing the posterior malleolus. This synergistic approach prevents re-dislocation while minimizing local soft tissue stripping, offering significant advantages in surgical technique selection (22–26).

## Limitations

This report aims to highlight the hidden entrapment pitfalls during Bosworth fracture-dislocation surgery and emphasize the critical value of standard lateral radiographs in assessing reduction quality through a retrospective analysis of a failed open reduction case. However, restricted by its single-case scope, short follow-up period, and retrospective design, the conclusions drawn herein should be interpreted as clinical warnings and practical references rather than high-level evidence-based medicine. Future prospective studies or multicenter case series are required to further establish the optimal surgical strategies and long-term prognoses for this complex injury.

## Conclusion

Bosworth fracture-dislocation is a rare and easily missed injury that requires a high index of clinical suspicion. Characteristic clinical and radiographic features, supplemented by computed tomography imaging, greatly aid in early diagnosis. Due to the mechanical difficulty associated with closed reduction, early open reduction and internal fixation are strongly recommended. Positioning the patient in a floating position provides excellent surgical exposure and facilitates essential intraoperative standard lateral imaging, thereby significantly reducing the risk of failed reduction.

## Data Availability

The original contributions presented in the study are included in the article/Supplementary Material, further inquiries can be directed to the corresponding authors.
